# Comparing future climatic suitability to shoreline loss for recreational beach use: a case study of five Japanese beaches

**DOI:** 10.1007/s10113-022-01906-2

**Published:** 2022-03-28

**Authors:** Andrew Zajch, Micah J. Hewer, William A. Gough, Keiko Udo

**Affiliations:** 1 Department of Physical and Environmental Sciences, University of Toronto at Scarborough, Toronto, Canada; 2 International Research Institute of Disaster Science, Tohoku University, Sendai, Japan

**Keywords:** Climate change, Impacts and adaptation, Holiday Climate Index, Sea-level rise, Beach tourism, Regional warming

## Abstract

Coastal tourism is impacted by regional environmental change, including sea-level rise and climatic change. A case study at five Japanese beaches was conducted to determine the relative difference in coastal and climatic changes that are important for recreational beach tourism. Future climate conditions and projections of shoreline changes due to sea-level rise were estimated using ensembles of 14 and 21 Global Circulation Models (GCMs), respectively. The Holiday Climate Index (HCI:Beach) specific to recreational beach use was used to assess historical and future climatic suitability while beach loss area was used to quantify physical changes to beach conditions. Diminished beaches with improved climate conditions were observed at all sites except Yonehara, which had both diminished beach and climate conditions. Comparing HCI:Beach scores relative to historical conditions revealed a change of −2.4 to +10%, while beach losses were typically >60%. These results highlight that beach tourism suitability at the study sites will likely exhibit greater changes due to sea-level rise, compared to those driven by changing atmospheric conditions.

**Supplementary Information:**

The online version contains supplementary material available at 10.1007/s10113-022-01906-2.

## Introduction

Global climate change is at the forefront of the challenges facing the tourism industry (Scott 2011). Japan’s travel and tourism industry in 2019 made up 7.1% of national GDP dropping by 37% in reaction to the COVID-19 pandemic in 2020 (World Travel and Tourism Council 2021). However, Japan’s domestic tourism industry positions Japan well for continued demand of tourism resources. Japan ranked 4th in domestic tourism spending for both 2019 and 2020, while observing a relatively low decrease in domestic tourism (~30%) compared to international tourism (~83%) in 2020, emphasizing that the future of tourism should not be ignored due its continued importance and engagement (World Travel and Tourism Council 2021). Studies have suggested that Japan’s high adaptative capacity is capable of minimizing the potential impacts of climate change to tourism (Perch-Nielsen 2010; Scott et al. 2019). By extension, it is important to identify priorities for adaptation to ensure tourism resources are maintained and managed effectively. The goal of this work is to compare the impacts of regional environmental changes from both beach loss and climatic suitability perspectives, to demonstrate the need for holistic assessments rather than the siloed approaches typically used.

Sandy beaches worldwide are threatened by sea-level rise (SLR) and related erosive processes stemming from global climate change (Vousdoukas et al. 2020). Unsurprisingly, SLR driven by climate change will lead to a reduction in beach area along Japanese coasts (Mori et al. 2018; Udo and Takeda 2017; Yoshida et al. 2013). However, the changes will not be homogenous across Japan. Mori et al. (2018) showed a decrease in beach widths in Japan, with 10–45% of beaches potentially losing more than half of their area depending on the degree of SLR projected by future climate change scenarios. An analysis of mobile phone data and SLR described considerable degradation of beach resources, particularly in the southern regions of Japan (Kubo et al. 2020). SLR impacts are expected to alter the state of recreational beaches in Japan, important resources for the tourism industry.

Such changes will directly impact beach tourism due to the reductions in physical capacity and tourist perceptions. In a case study of three beaches in Thailand, Ritphring et al. (2018) estimated a reduction in physical carrying capacity of beaches due to a reduction in beach width. As beach width is reduced, the physical limits on how many users the beach can comfortably accommodate decrease. Therefore, while the number of tourists may vary at a beach due to a multitude of factors, such as access or marketing, beach width provides a physical constraint on the potential useability of the tourism resource. Similar consequences of reduced beach width due to SLR were predicted from investigations in the Catalan region and the Caribbean (López-Dóriga et al. 2019; Scott et al. 2012a, b). Beach width can also play a factor in tourist perceptions. A case study from Mallorca found that beach width had the largest impact of visitor well-being compared to the loss of seagrass and an increase in beach closures due to jellyfish (Enríquez and Bujosa Bestard 2020). Therefore, beach loss and SLR have the potential to reduce recreational beach resources and negatively affect beach tourism in Japan.

To evaluate beach suitability for recreation and tourism, it is essential to understand the factors that influence tourist preference, perception, and experience. While the work by Kubo et al. (2020) illustrated the economic effects of climate change impacts on beach tourism relative to coastal changes, the authors recognized the need for the inclusion of atmospheric variables in future studies. A case study in Ontario, Canada, contrasting a coastal beach park to an inland forest park found that visitors to the beach were more likely to leave the park with the onset of extreme weather events (heavy rain and strong wind), and had a greater desire for comfortable temperatures and clear skies (Hewer et al. 2015). Uyarra et al. (2005) examined tourist opinion at two Caribbean destinations: Bonaire and Barbados, identifying weather and sea conditions as dominant factors in the tourists’ choice of destination. A case study in the UK determined warm weather would be a dominant driver of recreational beach use, particularly for sunbathers and paddlers, although changing beach width may have ancillary impacts on coastal environments and their recreational use (Coombes and Jones 2010). These examples demonstrate the intrinsic dependence of beach activities on weather conditions. Temperatures, and by extension thermal comfort, have often been investigated and correlated with beach tourism. For example, a survey in New South Wales, Australia, reported that sunbathing and swimming increase with higher air temperatures (Provost et al. 2019). However, de Freitas (2003) demonstrated that physical elements of climate (such as rain and wind) can have an overriding effect on tourist satisfaction and behavior in a beach tourism context, despite the presence of favorable thermal conditions. Therefore, to quantify climatic suitability for tourism, tourism climate indices have been developed combining several atmospheric variables which have a combined effect on the visitor experience. However, the evaluation of beach tourism, if engaged from strictly a climate index perspective, is often done without consideration of climate-induced environmental changes such as coastal erosion and beach width loss due to SLR (Moreno and Amelung 2009; Demiroglu et al. 2020).

Tourism climate indices provide a single metric for stakeholders which can describe the climatic suitability of a location based on historical weather conditions as well as future climate conditions using climate model projections. The Tourism Climate Index (TCI) is the most commonly used climate index for tourism, which incorporates thermal comfort, precipitation, sunlight hours, and wind speed into a single metric of favourability (Mieczkowski 1985). The TCI has been used for predicting climatic suitability of tourism destinations on global (Amelung et al. 2007) and regional (Scott et al. 2004) scales, including in Japan (Kubokawa et al. 2014). Improving on the goal of the TCI, different climate indices have since been developed, such as the Climate Index for Tourism (CIT) by de Freitas et al. (2008) and the Holiday Climate Index (HCI) by Scott et al. (2016). The CIT improved on the TCI as it incorporated the effects of physical thresholds, such as very strong winds and heavy rains which would “override” favorable thermal comfort conditions, while addressing the subjective “expert-opinion”–based weights assignment to the various climate components (de Freitas et al. 2008).

However, recreational beach use and beach tourism are predictably governed by a different regime of climatic preferences when compared to general tourism activities. A case study of the thermal preferences of tourists in the Caribbean showed that urban/indoor thermal comfort ranges cannot be applied to beach tourism (Rutty and Scott 2015). Due to the contrasting nature of beach activities and preferences, beach-specific indices were developed. Morgan et al. (2000), for example, devised a beach-specific climate index that included sea temperatures in addition to atmospheric conditions. There is also a beach-specific HCI that addresses the differences between beach users and urban tourists by increasing the importance of cloud cover and decreasing the weight of thermal comfort within the index (Matthews et al. 2019; Rutty et al. 2020). This index provides a pathway for better understanding the weather sensitivity of beach tourism and for assessing climate change impacts on the climatic suitability of such destinations.

There has been a paucity of work addressing climate change impacts on the physical environment for beach tourism, which would combine both atmospheric changes and climate-induced environmental changes (such as SLR). Demiroglu et al. (2020) presented a thorough projection of HCI indices for the Mediterranean, although this study did not include impacts of SLR. In the Japanese context, Kubokawa et al. (2014) conducted a regional climate change impact assessment using the TCI which is not specific to beach use. Similarly, Perch-Nielsen (2010) presented a beach tourism vulnerability index that examined the impacts of weather changes (again, using the TCI), and SLR for a global analysis. A regional study in East Anglia projected increased number of visitors due to higher temperatures, with beach width reductions having minimal effects (Coombes et al. 2009); however, the area was predicted to retain most of its historical beach width unlike the projections for the Japanese coastline (Udo and Takeda 2017). Therefore, this work aims to build upon these previous studies to compare the relative change in beach loss with climatic suitability described by HCI:Beach scores. HCI:Beach has been validated in both Canadian and Caribbean contexts, making it generally applicable to the Japanese context since the climate in Japan varies drastically from north to south: being characterized by conditions similar to Canada in the north (humid continental climate), and conditions similar to the Caribbean in the south (subtropical climate).

Understanding climate change impacts to recreational beaches is essential for directing adaptations. Beach nourishment is a popular approach, particularly for recreational beaches, to increase the width and volume of beaches by filling them with external supplies of sand (de Schipper et al. 2021). Yoshida et al. (2014) highlighted the higher cost of maintaining beaches for recreation relative to ecosystem or shoreline protection in Japan, due to the required larger width of the beach. The cost of adapting to beach losses through sand infilling justifies the development of derivable metrics on a regional scale for the identification of optimal candidates during site selection (Yoshida et al. 2014; Somphong et al. 2020). While the use of beach nourishment provides a means to protect the coast, it may not necessarily be a long-term strategy to adapt to SLR (Bongarts Lebbe et al. 2021). Incorporating climate suitability and beach loss together into one assessment methodology will help better understand the multi-faceted impacts and projected limiting factors introduced by climate changes. This is designed to help inform more comprehensive long-term adaptation strategies for recreational beaches in Japan. The methodology developed in this study may also be applicable for other locations across the globe and could therefore lead to greater understanding of climate change impacts on international beach tourism destinations.

## Methodology

### Site selection

**Fig. 1:**
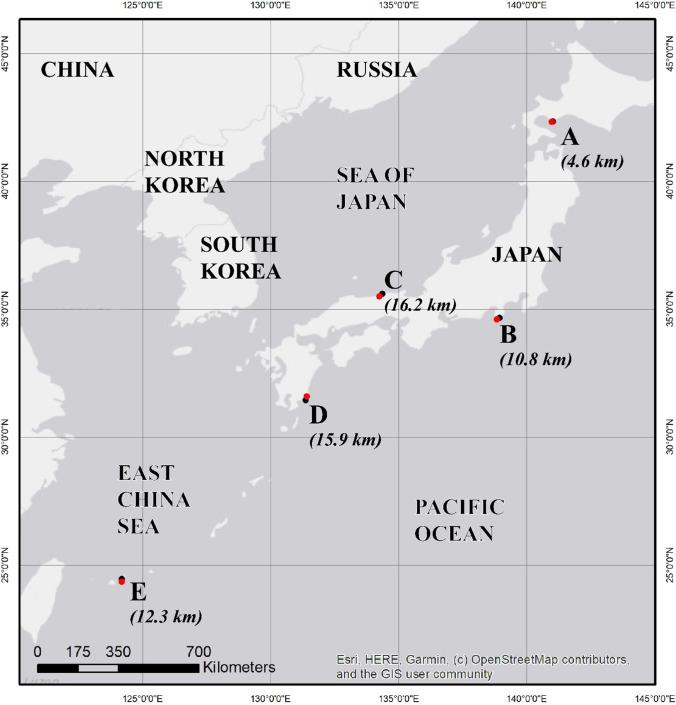
Map of the weather stations (red circles) and study sites (black circles) with distances between them shown in the brackets below the pairs. A) Muroran Station and Itanki Beach; B) Irozaki Station and Tatadohama Beach; C) Tottori Station and Higashihama Beach; D) Abaratsu Station and Ishinami Beach; and E) Ishigaki Station and Yonehara Beach. This figure was made in ARCGIS

Five beaches were chosen to capture a range of climates and ocean conditions experienced across Japan (Fig. 1). These sites not only represented different environments but were also adjacent to weather stations with the necessary atmospheric variables and a sufficient time series of observational data required for the analysis. The most northern location is Itanki Beach on the southern tip of Hokkaido (site A, Fig. 1). Tatadohama Beach (site B) is located on the southern tip of the Izu Peninsula. Higashihama Beach (site C) is in the Tottori prefecture along the coast of the Sea of Japan. While these latter two beaches have comparable latitudes, they are distinguished by their contrasting coasts, one on the Pacific Ocean and the other on the Sea of Japan. The most southern locations are Ishinami Beach (site D) on the southeastern coast of Kyushu, and Yonehara Beach (site E) on Ishigaki Island.

### Climatic suitability

#### Historical data

Daily weather data was downloaded from the five weather stations in Fig. 1 for the period of 1986 to 2005 (Japan Meteorological Agency 2020). This data consisted of total precipitation, daily maximum temperature, humidity, wind speed, and cloud cover. The daily weather data was filtered based on missing data to produce complete daily data points. Days with one or more missing parameters were removed from the historical data set prior to calculating the HCI:Beach index scores. A tabulation of total monthly days and the percent of available data days for each of the five beaches showed that all stations had at least 84% of days available for all months (Supplementary Materials – Table 1). Tatadohama was characterized by the greatest degree of missing data (~84–90% of days available), while Higashihama had a nearly complete daily data record used in the analysis.

The combination of weather variables retrieved allowed for a calculation of the HCI:Beach index (Eq. 1), following the approach of Rutty et al. (2020).

1 $$HCI: Beach=2\ (TC)+4(A)+\left(3(P)+W\right)$$

Each of the components in Eq. 1 (*TC*, *A*, *P*, *W*) was assigned a ranking based on thermal comfort (*TC*), aesthetic (*A*), precipitation (*P*), and wind speed (*W*), as detailed in Rutty et al. (2020). “*TC*” was based on the Humidex value which is a Canadian standard heat index (Matthews et al. 2019; Rutty et al. 2020). The Humidex was calculated using the *ThermIndex* package in *R* (Castelhano 2017). “*A*” was described by cloud coverage. The cloud coverage percentage was used for this value, calculated by converting the recorded value on a 10-point scale where 10 was 100% and 0 was 0%. “*P*” measured in millimeters and “*W*” recorded in km/h (converted from m/s) were used to determine the total precipitation and average wind speed rankings. It is important to note that only rainfall was considered for this analysis, with “*P*” not including snowfall.

#### Future projections

Future climate scenario data was downloaded from the World Climate Programmes Climate Model Intercomparison Project version 5 (CMIP5) for three representative concentration pathways (RCP2.6, RCP4.5, and RCP8.5), from 14 Global Climate Models (GCM) using the r1i1p1 ensemble (Appendix Table 1) (ESGF 2020). Monthly average daily maximum air temperature, relative humidity, and cloud area fraction were downloaded for historical (1890–2005) and future (2005––2100) periods. Historical observations at weather stations used for the climate baseline were not selected past 2005 to ensure the historical weather data sets represented time periods consistent with the historical simulations of the GCMs (monthly averages presented in Supplementary Materials Table 2).

Changes between historical baselines and future periods for each variable were tabulated by comparing the mean monthly values between the GCM’s historical and future data sets. This was done for the end of the twenty-first century only (2081–2100), for all three RCPs. The change estimated by each model was then superimposed on the daily historical conditions for the corresponding month, creating a set of future conditions for each GCM and each RCP. Manipulation of GCM data was done using the *Climate Data Operators* package (Schulzweida 2019). This was in addition to packages in the *R* Environment, which were used to manipulate, manage, and graph data sets (Auguie 2017; Pierce 2017; R Core Team 2018; Wickham et al. 2018a, 2018b; Wickham 2018).

Using a full ensemble approach (Hewer et al. 2016), which averages the outputs for all 14 GCMs, a set of future climate conditions were produced for each RCP and GCM. This ensemble was limited to model outputs which had the necessary data for all three RCP scenarios investigated. Changes in surface air temperature, relative humidity, and cloud cover derived from the ensembles were applied to the daily weather station data. These change factors were based on the daily maximum air temperature (*tasmax*), relative humidity (*hurs*), and cloud cover (*clt*) variables from CMIP5. The super-imposition of changes, either absolute or percentage, attempted to emulate the “morphing” approach presented by Belcher et al. (2005) for evolving historical conditions to a representation of future conditions; albeit this study did so on a daily scale rather than an hourly scale. The absolute difference in surface air temperature (°C) between the historical baseline and future conditions for each month was applied to every daily data point for the appropriate month. The absolute difference in humidity (%) and cloud cover (%) was applied to historical values to produce future conditions. Cloud cover required a conversion of the cloud cover (%) described by the GCMs into a 10-point scale, consistent with the historical scale, prior to applying the change. In the case of both humidity and cloud cover, values defying logical thresholds (<0 or >10 for cloud cover, or >100 % for humidity) were automatically set to the surpassed threshold. Using these future climate change scenarios, the HCI was calculated on a daily timescale. The median monthly value for each scenario, time period, and model was then used to produce a database from which the intermodal median, maximum, and minimums can be extracted. It should be noted that the wind speed and precipitation variables were not altered as their dependent weather parameters were not estimated for future conditions. This can be justified by the optimized HCI:Beach index weightings which revealed lower weights for precipitation ratings and an omission of wind entirely (Matthews et al. 2020).

### Beach loss

The projections of shoreline changes were based on previous studies (Udo and Takeda 2017; Takeda and Udo 2020), which used 21 GCMs for a range of RCP scenarios and sediment sizes to estimate beach loss across Japan (see Appendix Table 1). The GCM ensemble data is presented in the IPCC’s Fifth Assessment Report (Church et al. 2013a, 2013b). These projections were done using Bruun’s rule, which enable the estimate of the change in sandy beach/shoreline profiles for different SLR scenarios (Eq. 2) (Bruun 1962). Bruun’s rule has been used previously to estimate beach loss in the context of tourism within the Caribbean sea (Scott et al. 2012b), as well as along the coast of Thailand (Nidhinarangkoon et al. 2020).

2 $$\frac{\Delta y}{y_{\ast }}=\frac{-S}{h_{\ast }+{B}_h}$$

The degree of shoreline regression (Δ*y*) is described as a function of sea-level rise (*S*), while beach characteristics are defined by the depth of closure (h_*_), horizontal distance to the depth of closure (y_*_), and berm height (*B*_*h*_) (Udo et al. 2016). The depth of closure is derived using Eq. 3, as illustrated by Nicholls et al. (1996), while berm height is estimated by Eq.4, as presented by Takeda and Sunamura (1983). *H*_*e,t*_ represents the significant wave height threshold that is only surpassed for 12 h over a set amount of years, *t*, which is 60 years in this study. *T*_*e,t*_ corresponds to the wave period for *H*_*e,t*_. The breaking wave height (*H*_*b*_) and mean significant wave period (*T*_*s*_) are used to calculate berm height in Eq. 4 (Udo and Takeda 2017).

3 $${h}_{\ast }=2.28{H}_{e,t}-68.5\left(\frac{H_{e,t}^2}{g{T}_{e,t}^2}\right)$$

4 $${B}_h=0.125{H}_b^{5/8}{\left(g{T}_s^2\right)}^{3/8}$$

The breaking wave height (Eq. 5) is derived from the formulation provided by Sunamura (1983), which describes it as a function of mean significant wave height (*H*_*s*_), the beach slope represented by tan*α*, and the mean significant wave length, *L*_*s*_.

5 $$\frac{H_b}{H_s}={\left(\tan \alpha \right)}^{0.2}{\left(\frac{H_s}{L_s}\right)}^{-0.25}$$

The values employed for this approach are consistent with the work by Udo and Takeda (2017). The slope and sediment diameter were assumed to be 0.0322 and 0.3 mm, respectively, with the latter being based on a review of Japanese beaches (Udo and Takeda 2014). Wave height data was derived from the 3-h significant wave dataset of the Japanese Meteorological Agency’s Coastal Wave model for the period of 2005 to 2009 (Tauchi et al. 2007). *H*_*e,t*_ was the maximum significant wave height and *H*_*s*_ represented the 5-year average (Udo and Takeda 2017). Ultimately, the estimate of shoreline retreat was determined using Eq. 2 and compared against the historical beach width from 1990, to project beach loss at the sites. The original shoreline width is computed from historical beach area and beach length, presented in Udo et al. (2016). It should be noted that it is assumed that shoreline retreat cannot extend beyond the historical limit (Udo and Takeda 2017).

### Relative importance of changes

In order to examine the relative change in beach properties between shoreline changes and the HCI:Beach index, a comparison metric was devised. The percentage difference between future and historical conditions (∆*X*_*future* − *historical*_) was scaled based on the historical median (*X*_*historical*, *median*_), determined from the weather station data (Eq. 6). The percentage difference will be larger for cases when changes perturb the historical baseline conditions. In the case of beach loss, the percentage loss of beach area is relative to the historical baseline, as expressed in Eq. 6. This allows changes to the HCI:Beach index and beach loss to be compared on the same scale. It should be noted however that %Δ*X* will be naturally larger for variables with low historical magnitudes, and therefore, the metric should be viewed as a measure of the magnitude of deviation from historical conditions.

6 $$\%\Delta X=\frac{\Delta {X}_{future- historical}}{X_{historical, median}}$$

The large uncertainty of beach width change due to SLR in Japan has motivated the use of a metric that accounts for the range of potential outcomes derived from climate change scenario ensembles (Kubo et al. 2020; Udo and Takeda 2017). This is a result of the range of SLR scenarios predicted by climate models and sediment size which cannot be addressed significantly with an increase in spatial resolution (Takeda and Udo 2020). This is not an issue unique to beach conditions. Projections of climate indices are also subject to the inherent challenge of diverging GCM or Regional Climate Model (RCM) outputs, resulting in different conclusions (Dubois et al. 2016). Therefore, the changes to beach width and climate indices should also include a description of the changes within the domain dependent on the results of the GCM range. A comparison of the model range was conducted for the RCP4.5 scenario exploring only the 14 GCM models used for climatic analysis. This is a smaller subset than the 21 GCM ensemble used for SLR projections (Church et al. 2013b). The difference between maximum (%Δ*X*_*future,max*_) and minimum (%Δ*X*_*future,min*_) for percentage change in HCI:Beach scores or beach loss from the 14 GCMs was used as a metric to describe the range of projections (Eq. 7). The results were not standardized due to the skewness of the beach loss data.

7 $$\%\Delta {X}_{range}=\%\Delta {X}_{future,\mathit{\max}}-\%\Delta {X}_{future,\mathit{\min}}$$

## Results

### Historical climatic suitability

**Fig. 2:**
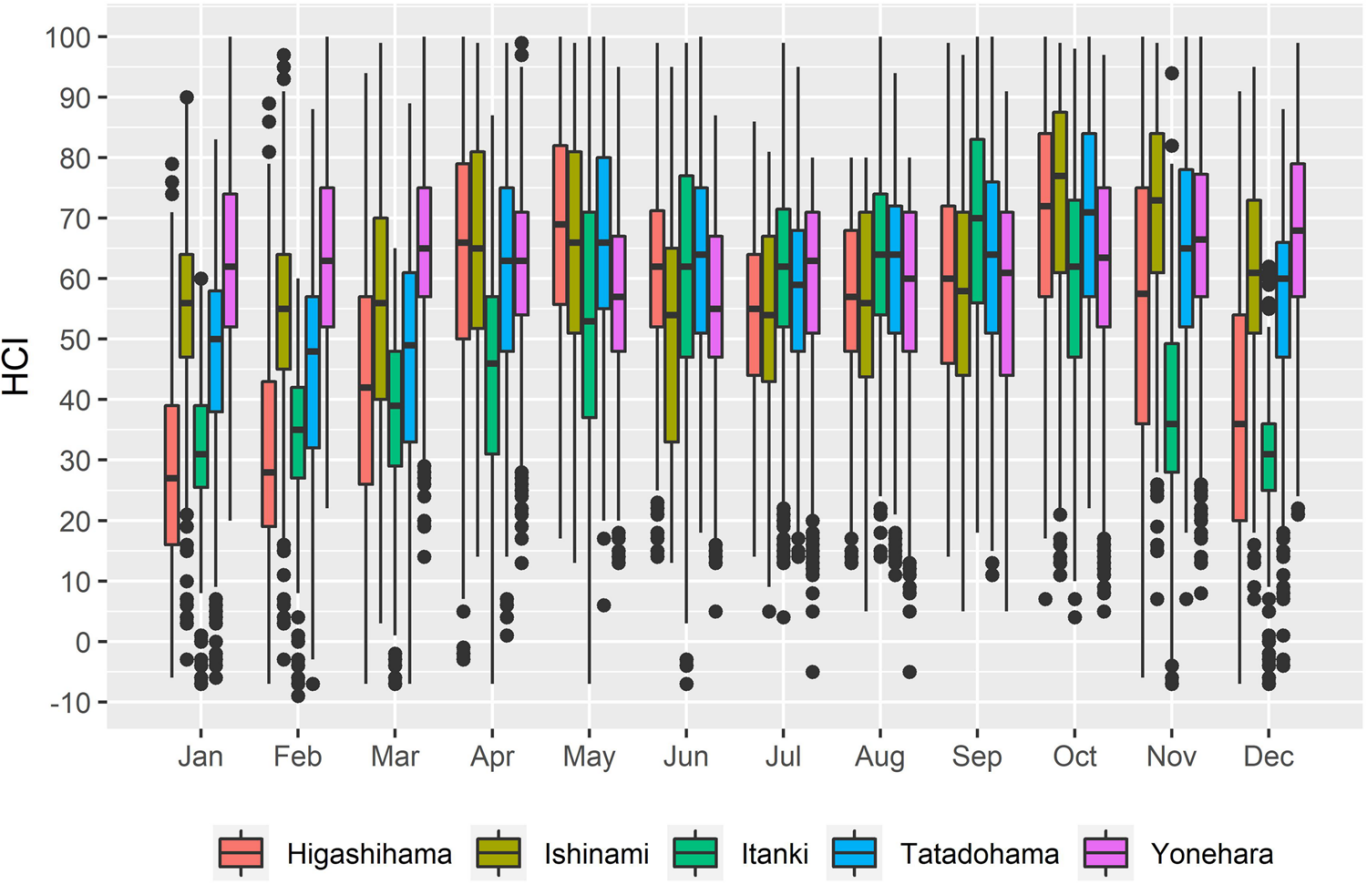
Monthly HCI:Beach scores for the five beaches investigated. Each site is represented by a boxplot showing the median as well as range of daily HCI values calculated for each month. Dots represent possible daily HCI outliers while colored sections identify the interquartile range about the median, identified by the center line of the box

HCI:Beach scores vary seasonally and among sites (Fig. 2). As expected, the lowest HCI:Beach scores are reported during winter months (December to February), with a noticeable exception at Yonehara and Ishinami, which maintain index values between 55 and 68, even during the winter, and lows of 54–55 during spring/summer months. Yonehara has a much lower seasonal variability in HCI:Beach scores, whereas Higashihama, and to a lesser degree Itanki, record large changes from winter to summer seasons. During the winter months, the HCI:Beach score was <36 for Itanki and Higashihama. HCI:Beach scores peaked during the summer and fall (June to October), for Tatadohama, Itanki, and Higashihama beaches, while Yonehara and Ishinami recorded the highest values in the fall and early winter (September to December). HCI:Beach scores at Ishinami also had a noticeable increase in the spring, prior to a decrease during the summer months. The highest median HCI:Beach score across the five sites was observed in October at Ishinami beach, with an approximate value of 77. A similar peak in October was observed for Higashihama and Tatadohama, with HCI:Beach scores of 72 and 71, respectively. Itanki’s peak score of 70 was observed in September. Ultimately, the HCI:Beach scores demonstrate a seasonal dependence on climatic regimes with cooler climates observing a greater degree of seasonality. The mechanisms for the contrasting behavior between sites is explained by the seasonal and site-specific variability in the atmospheric variables used to calculate HCI:Beach scores.

**Fig. 3:**
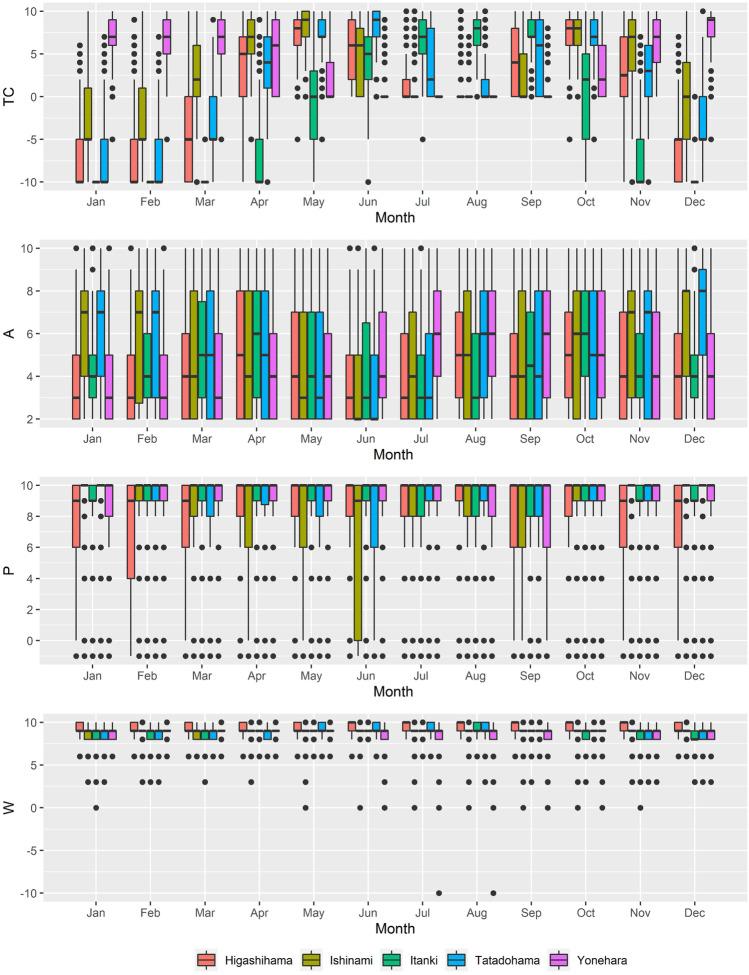
Boxplots of the daily ratings of the thermal Comfort (*TC*), aesthetic (*A*), precipitation (*P*), and wind (*W*) components of the HCI:Beach scores for each month and site. Dots represent possible daily parameter outliers while colored sections identify the interquartile range about the median, identified by the center line of box

Thermal comfort displays the greatest degree of variability across sites and months explored (Fig. 3). Yonehara recorded the most consistent thermal comfort attributable due to its less pronounced seasonality. It has its highest thermal comfort values from November to March. During the summer months, thermal comfort is roughly 0 due to high humidex values. At the other beach sites, except Itanki, the period from April to June and September to November had the highest thermal comfort values. Positive thermal comfort is confined more to summer months for the coldest site, Itanki. Compared to thermal comfort, the degree of variability shown in the aesthetic, precipitation, and wind parameters was minimal. Median aesthetic is typically less than 8, with a noticeable low aesthetic period in June. This coincides with high precipitation (P) at Ishinami and Tatadohama during this month. Low aesthetic values are accompanied by synchronous low P values at Higashihama, Itanki, and Ishinami throughout the year. However, median *P* values are typically ≥9, revealing that precipitation is not typically a limiting factor. In terms of the wind parameter (*W*), there was minimal monthly variation, with lower ratings occurring throughout winter months. Therefore, *W* was not a prominent factor for varying HCI:Beach scores.

**Fig. 4:**
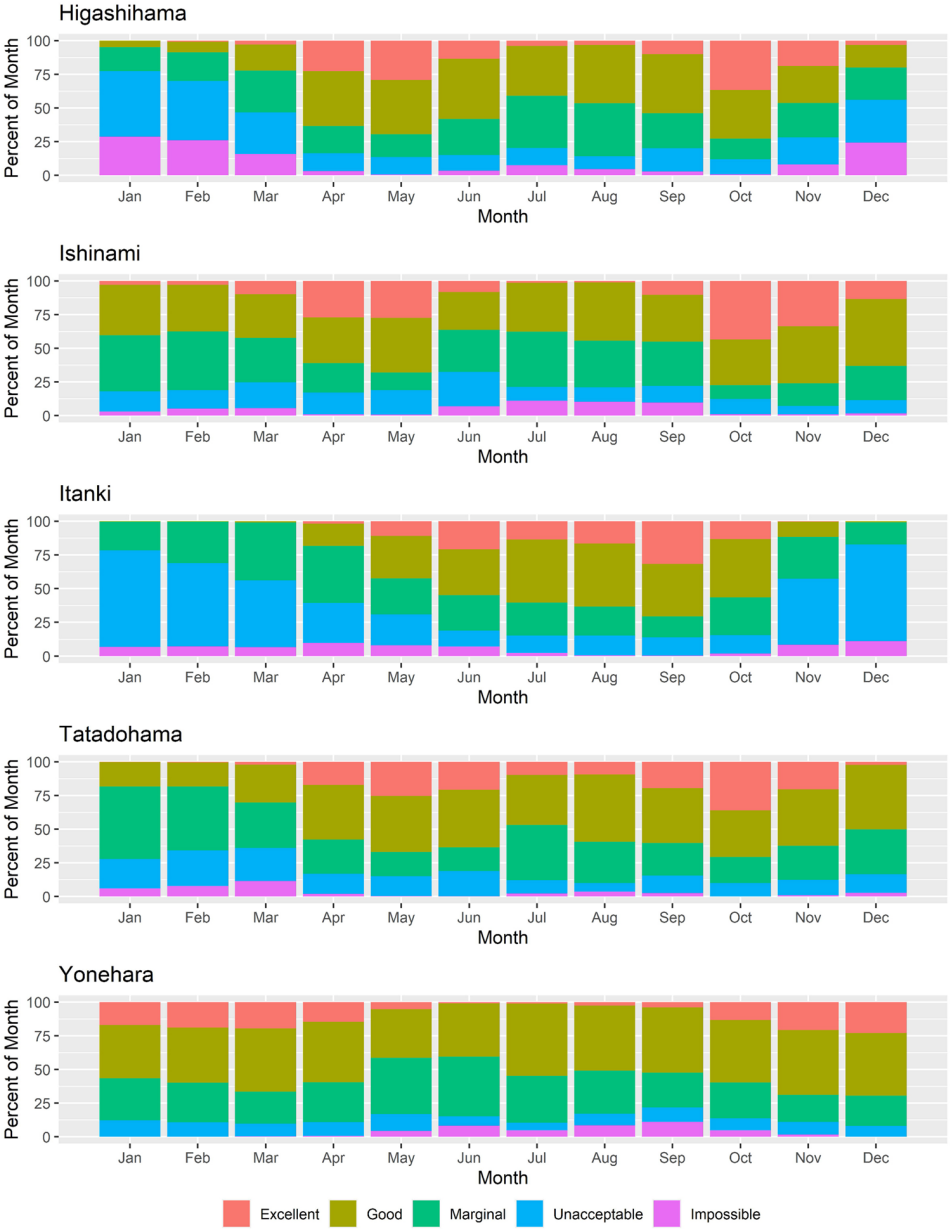
The percentage of occurrence for varying HCI:Beach score classifications for each month based on the categories described in Rutty et al. (2020): Impossible (0–19), Unacceptable (20–39), Marginal (40–59), Good (60–79), and Excellent (80–100)

Classifying historical HCI:Beach scores highlighted the seasonal regime in beach suitability (Fig. 4). Yonehara observed roughly 20% “excellent” suitability from November through March. The loss of “excellent” beach suitability throughout the summer months at Yonehara was also observed at Ishinami, Tatadohama, and Higashihama, that instead had peaks in “excellent” beach suitability from April to June and October to November, with values >15%. Higashihama and Itanki had the highest rate of “unacceptable” and “impossible” conditions extending from fall to spring months; being particularly persistent in the cooler Itanki, but more pronounced in Higashihama. These classifications reinforce the stability of climatic suitability at Yonehara, and the natural seasonality at other, particularly cooler, sites.

### Comparing climatic suitability and shoreline changes

**Fig. 5:**
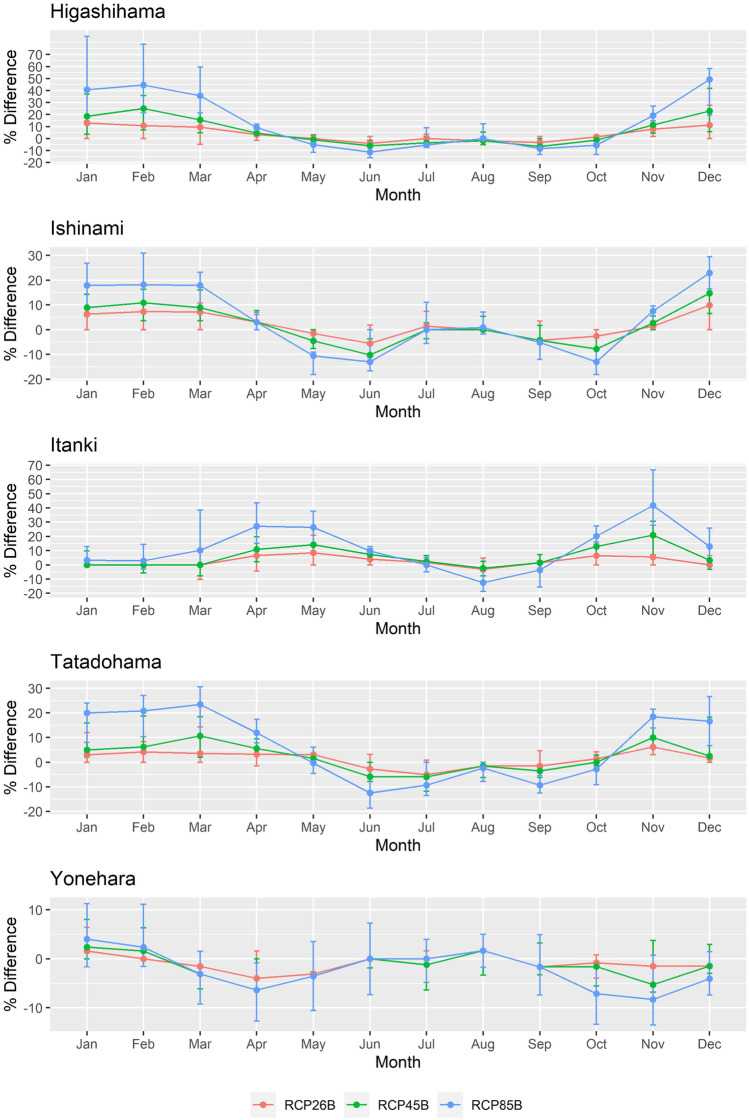
Percent difference of HCI:Beach scores for the five locations comparing projected and historical values. Larger magnitudes represent greater changes relative to historical values. The central dots represent the median of the models used while the upper and lower limits illustrate the maximum and minimum changes estimated by distinct GCM models

A percent difference (as seen in Eq. 6) of projected HCI:Beach scores compared to historical conditions demonstrated the relative difference in climatic suitability predicted for the five beaches (Fig. 5). Generally, months with high favorability suffered reductions in HCI:Beach scores under projected climate change. Meanwhile, periods where HCI:Beach scores were unfavorable, due to low thermal comfort, witnessed positive percent differences. Higashihama, Ishinami, and Tatadohama observed improvements from November to April, while Itanki showed improvements in all months except August and September. This represented increases in beach tourism favorability from a climate perspective, although this does not necessarily imply suitability. For example, low percent differences at Yonehara, Ishinami, and Higashihama during the summer were due to the saturation of the unsuitable thermal comfort which could not decrease further. As a result, the largest changes occur at sites where there is still capacity for thermal comfort indices to reduce further. It is apparent that the occurrence of negative percent difference values is being driven by decreasing thermal comfort in historically high humidex periods. Therefore, cooler sites tend to benefit more from projected climate change scenarios with improvements in thermal comfort. In terms of scenarios, the magnitude of the percent difference change was greatest for RCP8.5, which can be interpreted as the highest emissions scenario with the greatest degree of radiative forcing by 2100. RCP8.5 had the largest range in percent differences within the domain of GCM results. RCP2.6 showed the lowest changes, as this scenario represents a mitigation pathway with reduced emissions and lower radiative forcing by the end of the century. The dynamics between varying RCP scenarios are expected and follow the behavior of estimated beach loss.

**Fig. 6:**
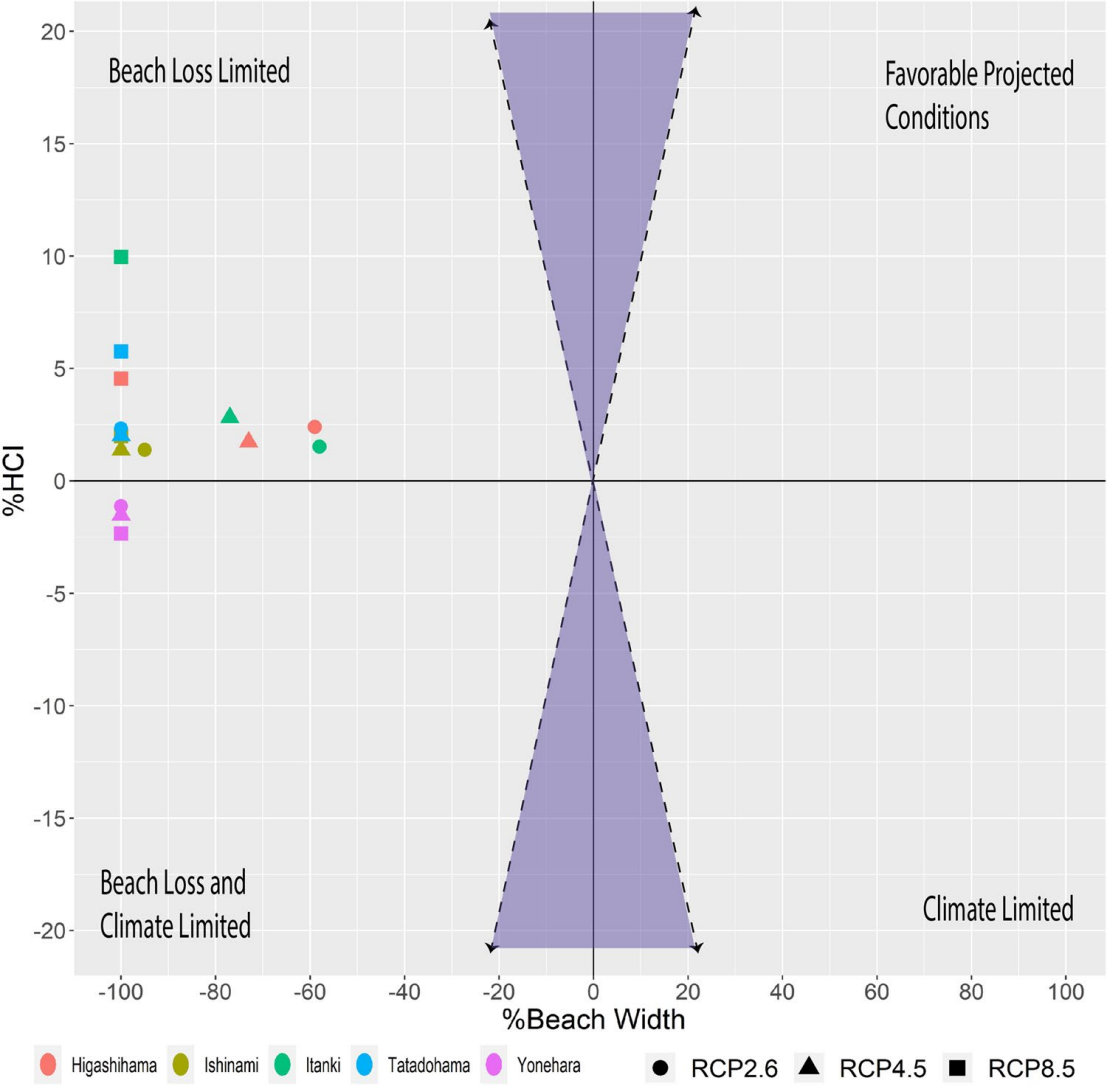
Relative difference in annual HCI:Beach scores, generated by calculating the median of monthly HCI:Beach score changes, and beach width for the 5 sites and 3 RCP scenarios. The domain can be broken down into 4 sectors representing the outcome of projected conditions. The shaded area represents where HCI:Beach score changes are greater than projected beach width changes (%), although none of the results is characterized by this behavior

Comparing beach loss (%) against the percentage change in HCI:Beach scores demonstrates the relatively larger projected changes in physical beach conditions (Fig. 6). In terms of climatic suitability, on an annual scale, all the sites except Yonehara are projected to experience positive changes. The range of values between RCP scenarios for climatic suitability is greatest at cooler sites (Higashihama, Tatadohama, and Itanki). Similarly, the greatest changes are observed for RCP8.5 for Tatadohama and Itanki, with both sites showing changes >5%. However, compared to beach loss, the magnitude of these changes is minimal as beach loss at all sites is >50% under all emissions scenarios and all sites converge at 100% beach loss for RCP8.5 by the end of the century. Higashihama and Itanki beaches do exhibit variation among the RCP scenarios and therefore observe the lowest beach loss values for RCP2.6 and RCP4.5. Yonehara and Tatadohama were the only sites which projected a beach loss of 100% for all scenarios. This is understandable since they have the smallest historical beach widths.

The range of beach loss and HCI:Beach score changes from 14 GCMs for the RCP4.5 scenario were also explored to understand the domain of outcomes (see Appendix Table 1 for GCMs used). In terms of magnitude, the range of beach loss estimates was greater than those observed for improved HCI:Beach scores. While this is likely a function of the magnitude of the percent changes of beach width (beach loss), it nevertheless shows that while beach loss estimates are greater, they are also subject to greater uncertainty in terms of the GCM model domains. Yonehara had the lowest range for beach loss (34.9%) and the lowest mean monthly range of HCI:Beach scores (6.9%). The largest range in beach loss projections occurred at Ishinami (66.7%), while Higashihama had the largest range in HCI:Beach score changes (16.9%). The sites with the greatest degree of beach loss variability were all located on the Pacific Coast of Japan, excluding Yonehara and Tatadohama. The low beach widths at Yonehara (~20 m) and Tatadohama (~30 m) resulted in a rapid convergence to 100% beach loss among models, reducing inter model variance. HCI:Beach score ranges reflected the magnitude of score changes with Higashihama and Itanki having the greatest ranges.

## Discussion

The results of this work suggest that the impacts of sea-level rise will have a greater effect on beach tourism resources than changes in climatic suitability. Based on projected changes, the magnitudes of beach loss (>50%) indicate that beach loss will be severe on Japan’s coasts. The large ensemble range (~35–65%) emphasizes the sensitivity of beach loss estimates and the need for more localized projections. Meanwhile, HCI:Beach scores on an annual scale are modified in the range of −2.4 to +10%. Even on a monthly scale, despite much higher values projected relative to the annual monthly median, changes are still typically<40% and below the lowest projected beach loss. However, the predominance of climatic suitability changes in fall and spring seasons, shortening or extending the potential recreational beach use season, amplifies the importance of HCI:Beach score changes. Ultimately, the results demonstrate the diverging narratives prescribed by projected beach loss and climatic suitability changes warrant the use of a multi-faceted approach to climate change impact assessments on beach tourism.

Contrasting projected beach loss and climatic suitability changes provide a qualitative framework for informing adaptation. All sites except Yonehara were characterized as only being limited by beach loss. These sites present opportunities for beach nourishment and broader climate change adaptation to help preserve the physical extent of the beach as climate suitability will improve, thus enhancing the useability of the beach from a physical limitation perspective. Yonehara could also benefit from beach nourishment although the framework reveals it will be both climate and beach loss limited. However, reductions in HCI:Beach scores are predominantly observed during spring and fall months when Yonehara has the highest incidence of “Excellent” conditions. Despite the decrease in climatic suitability, Yonehara’s historically high suitability is still dominated by “Good” conditions. Further localized analysis is required to account for the sites’ nuances and tourist visitation patterns to supplement the framework and to establish the cost-effectiveness of beach nourishment. Nevertheless, the analysis demonstrates that avoiding a siloed approach to climate change impact assessment of beaches presents both the opportunities and challenges which stakeholders need to be cognizant of to establish effective adaptation plans.

By classifying the relative changes in the climate index and beach width, an outcome framework can be presented which provides a straightforward representation for stakeholders. For example, if HCI:Beach scores and beach width both increase, then the “projected” conditions demonstrate an improvement in favorability, while a reduction in both reflects the site being limited from both a climatic and physical perspective. However, if a site only displays a reduction in HCI:Beach scores, and an increase in beach width, then the site is only degraded by the suitability of the projected climate. On the other hand, a beach loss in conjunction with an increase in HCI:Beach scores shows that the suitability of the beach will be limited solely by coastal degradation. Categorizing the results in terms of their quadrants can highlight opportunities for adaptation. While prevailing climate conditions cannot be directly controlled, sites which are only beach loss limited are interesting targets for adaptation efforts (e.g., beach nourishment). For the five sites explored, all the sites except Yonehara are only beach loss limited. This implies that adaptation measures capable of mitigating the impacts of beach loss can help maintain or even improve the tourism resource potential of these locations.

### Limitations

The use of HCI:Beach was subject to inherent limitations. The composite ratings of the HCI:Beach scores were based on Caribbean (subtropical climate) conditions, introducing additional uncertainty due to potential regional and cultural differences in tourist climate preferences (Demiroglu et al. 2020; Matthews et al. 2019). Application of the index to southern sites, such as Ishinami and Yonehara, is most appropriate due to their similarity to Caribbean climates where the HCI:Beach index was calibrated and validated. However, the consistently low thermal comfort conditions at Yonehara throughout the summer suggest that modification of the comfort thresholds might be necessary to account for differing perceptions by local tourists. Comparing visitation numbers and weather conditions in the case study area could help optimize the weighting and improve efficacy of the HCI:Beach scores, as done by Matthews et al. (2019), in a Canadian context (humid continental climate), and Demiroglu et al. (2020), in a Turkish context. This analysis using HCI:Beach scores also does not address the potential impacts of projected climate changes at the locations of origin for the visiting tourists. A survey in the Caribbean revealed that tourists originating from temperate and tropical climates may have differences in ideal and unsuitable climate conditions (Rutty and Scott 2013). Therefore, the home climate that a tourist is accustomed to may influence their perception of climatic suitability at the beach. Furthermore, the importance of the “push” factor, based on tourists’ home climate, has been shown to be more important than the destination climate (Matthews et al. 2020). Climatic suitability based solely on the beach’s climate may require additional corrections to account for tourists’ home climate and perceptions unique to the study area.

The greater magnitude of beach loss relative to climatic suitability should be viewed while considering the assumptions of the approaches used in this work. The large ranges (~35–65%) in beach loss, compared to HCI:Beach scores (~7–17%), reveal a greater uncertainty in terms of projected beach conditions. Examining the sensitivity of beach loss estimates in Japan highlighted beaches with smaller sediments (0.2 mm) had larger expected impacts relative to conditions when larger sediments were assumed (0.6 mm) (Udo and Takeda 2017). The results presented in this study for beach loss may therefore be associated with greater impacts due to the assumed smaller sediment size (0.3 mm). It was also assumed that the landward extent of the beach was fixed. This may have the potential to overestimate beach loss if in reality the beach’s inland migration is not impeded by anthropogenic infrastructure or natural features (Cooper et al. 2020). In terms of the HCI:Beach scores, only the *TC* and *A* components were modified, omitting the physical parameters (*P* and *W*). Leaving *P* and *W* values unchanged may hide other climate-induced changes in the index. However, this was justified due to the relative stability or improvement of the aesthetic parameter, representative of cloud cover, implying that greater precipitation amounts should not be expected.

The recreational beach suitability results are also limited by the assumption that beach loss and climatic suitability are proportional in importance. This makes comparing a percentage change in beach loss and climatic suitability difficult since it does not consider the relative value of climatic suitability compared to beach width for the attractiveness of the tourism resource. This can also be amplified by the aggregation of weather phenomenon into a single daily index which may misconstrue the significance of a particular weather parameter. For example, precipitation can have a short temporal effect that does not necessarily influence the entire day (Scott and Jones 2007; Yu et al. 2009). Beach changes were calculated based on mean changes to sea conditions; as a result, analysis of sporadic, extreme events such as typhoons which may abruptly shift the beach profile, and can also have lingering effects on tourism, were not considered in this analysis (Gössling et al. 2012).

### Future work

To address some of the limitations of this work, future studies are proposed to provide a more comprehensive assessment of climate change impacts. Future work should look to account for extreme events, namely, probabilistic estimates of significant beach degradation that could discourage recreational use. For example, the sporadic erosion of a beach due to a storm. Aside from shoreline dimensions, there are other physical criteria which dictate the quality of the recreational beach resource. Future work should integrate such aesthetic factors of the beach environment. Some examples include water clarity and sediment color analysis, as done in the 3S (Sea, Sun, and Sand) analysis of Mestanza-Ramón et al. (2020), or scenic evaluation (Ergin et al. 2004; Iglesias et al. 2018). Such ancillary effects of projected climate change, including physical changes to the beach environment, have not been explored sufficiently (Enríquez and Bujosa Bestard 2020). Therefore, the results of this work should be viewed as a preliminary comparison of beach tourism vulnerability from climatic and physical perspectives, primarily highlighting limiting physical factors for future recreational beach use.

Future work is also necessary to better inform tourism climate indices. Validation for Japanese coastal tourism would be an instructive next step to calibrate the HCI:Beach score for local conditions. Tourists’ home climate can also impact their perception of climate suitability. For example, the impacts of climate change on thermal comfort for beach use may be offset among tourists living in cities experiencing urban heat island effects and reduced thermal comfort. Examining the relative change in climate conditions of tourists’ home environments and the beach destination’s climate is therefore an intriguing avenue for future research.

## Conclusion

The results of this work provide an insightful starting point for future investigations into the relative impact of projected climate change on recreational beach tourism resources in Japan and globally. This study highlights the value of assessing multiple impacts on beach tourism resources, rather than using the typical siloed approach common within the tourism literature. This is highlighted by the diverging conclusions from climatic suitability and beach loss perspectives, while projected climatic suitability implies a general improvement in recreational beach favourability; in contrast, projected beach loss reveals a degradation of recreational beach resources. The need for holistic climate change impact assessments considering both atmospheric changes and climate-induced environmental changes is emphasized for stakeholders evaluating beach tourism resources. The assessment framework demonstrated in this paper, which integrates both projected changes in physical constraints (beach width) and atmospheric conditions (beach-specific climate indices), can help communities better anticipate climate change impacts on beach tourism and inform priorities for adaptation.

## Supplementary Information

ESM 1 (DOCX 31 kb)

ESM 2 (DOCX 15.6 kb)
