# Supplementary material for: Comparing future climatic suitability to shoreline loss for recreational beach use: a case study of five Japanese beaches
Source: Reg Environ Change. 2022 Mar 28;22(2):54. doi: 10.1007/s10113-022-01906-2 (PMC8958812; doi:10.1007/s10113-022-01906-2)
Supplement: Supplementary file 1 — (DOCX 31 kb) [file 10113_2022_1906_MOESM1_ESM.docx]

**Supplementary Materials**

Table 1 - Available days (%) and total days evaluated for the baseline time period from 1986 to 2005 presented on a monthly basis for each study site.

| Month | Higashihama | | Ishinami | | Itanki | | Tatadohama | | Yonehara | |
| --- | --- | --- | --- | --- | --- | --- | --- | --- | --- | --- |
|  | % | Days | % | Days | % | Days | % | Days | % | Days |
| Jan | 99.83871 | 619 | 89.8387 | 557 | 99.838 | 619 | 89.354 | 554 | 100 | 620 |
| Feb | 100 | 565 | 89.9115 | 508 | 100 | 565 | 89.380 | 505 | 100 | 565 |
| Mar | 99.83871 | 619 | 90 | 558 | 99.838 | 619 | 89.677 | 556 | 100 | 620 |
| Apr | 99.66667 | 598 | 88.6666 | 532 | 99.833 | 599 | 90 | 540 | 100 | 600 |
| May | 100 | 620 | 89.1935 | 553 | 99.838 | 619 | 89.677 | 556 | 100 | 620 |
| Jun | 100 | 600 | 88.1666 | 529 | 99.833 | 599 | 90 | 540 | 99.6666 | 598 |
| Jul | 100 | 620 | 89.3548 | 554 | 99.838 | 619 | 90 | 558 | 100 | 620 |
| Aug | 100 | 620 | 89.0322 | 552 | 100 | 620 | 89.193 | 553 | 99.6774 | 618 |
| Sep | 100 | 600 | 89.1666 | 535 | 99.666 | 598 | 89.3333 | 536 | 100 | 600 |
| Oct | 100 | 620 | 85 | 527 | 99.838 | 619 | 84.193 | 522 | 99.6774 | 618 |
| Nov | 100 | 600 | 84.1666 | 505 | 100 | 600 | 85 | 510 | 100 | 600 |
| Dec | 100 | 620 | 84.6774 | 525 | 99.838 | 619 | 84.677 | 525 | 100 | 620 |

Table 2 – Historical and GCM average monthly historical values for daily maximum temperature (Tx, ⁰C), relative humidity (RH,%) and cloud cover (CC) as well as the differences between the two datasets.

|  | | | Months | | | | | | | | | | | |
| --- | --- | --- | --- | --- | --- | --- | --- | --- | --- | --- | --- | --- | --- | --- |
|  |  |  | 1 | 2 | 3 | 4 | 5 | 6 | 7 | 8 | 9 | 10 | 11 | 12 |
| Higashihama | Weather Station | Tx | 8.1 | 8.7 | 12.5 | 18.9 | 23.3 | 26.6 | 30.4 | 32.0 | 27.4 | 21.8 | 16.6 | 11.2 |
|  |  | RH | 75.5 | 74.0 | 70.0 | 66.6 | 69.2 | 74.1 | 76.4 | 74.1 | 77.8 | 76.2 | 74.6 | 74.2 |
|  |  | CC | 8.5 | 8.2 | 7.7 | 6.6 | 7.0 | 8.0 | 7.8 | 6.8 | 7.5 | 6.7 | 7.1 | 7.7 |
|  | GCM | Tx | 6.9 | 7.5 | 10.9 | 16.1 | 20.5 | 24.1 | 26.8 | 27.8 | 25.2 | 19.8 | 14.2 | 9.4 |
|  |  | RH | 81.7 | 80.2 | 78.1 | 78.1 | 81.6 | 86.6 | 88.5 | 86.6 | 84.0 | 80.4 | 80.9 | 81.6 |
|  |  | CC | 6.5 | 6.4 | 5.9 | 5.7 | 6.0 | 6.6 | 6.6 | 5.6 | 5.2 | 4.5 | 5.0 | 6.0 |
|  | Difference | Tx | 1.2 | 1.2 | 1.6 | 2.8 | 2.8 | 2.6 | 3.6 | 4.2 | 2.2 | 2.1 | 2.4 | 1.7 |
|  |  | RH | -6.2 | -6.1 | -8.2 | -11.6 | -12.3 | -12.5 | -12.1 | -12.5 | -6.3 | -4.2 | -6.2 | -7.4 |
|  |  | CC | 2.0 | 1.8 | 1.8 | 0.9 | 1.0 | 1.4 | 1.2 | 1.2 | 2.4 | 2.2 | 2.1 | 1.7 |
| IShinami | Weather Station | Tx | 13.7 | 14.3 | 16.8 | 20.8 | 24.1 | 26.6 | 30.6 | 31.1 | 28.6 | 24.5 | 20.0 | 15.8 |
|  |  | RH | 63.4 | 63.1 | 68.4 | 70.7 | 75.2 | 81.3 | 80.5 | 79.7 | 77.6 | 72.7 | 70.2 | 66.4 |
|  |  | CC | 4.9 | 5.5 | 6.6 | 6.7 | 7.4 | 8.4 | 7.1 | 6.5 | 6.8 | 5.8 | 5.4 | 4.7 |
|  | GCM | Tx | 12.9 | 13.0 | 15.2 | 18.6 | 21.9 | 25.0 | 27.3 | 28.2 | 26.8 | 23.2 | 19.0 | 15.2 |
|  |  | RH | 75.5 | 74.1 | 73.8 | 76.9 | 81.4 | 86.2 | 85.5 | 82.4 | 79.0 | 74.2 | 73.7 | 75.3 |
|  |  | CC | 5.1 | 5.0 | 5.1 | 5.7 | 6.2 | 6.8 | 6.2 | 5.3 | 4.7 | 4.2 | 4.4 | 4.9 |
|  | Difference | Tx | 0.9 | 1.3 | 1.6 | 2.2 | 2.2 | 1.6 | 3.3 | 2.8 | 1.8 | 1.2 | 1.0 | 0.6 |
|  |  | RH | -12.1 | -11.0 | -5.4 | -6.1 | -6.2 | -4.9 | -5.0 | -2.7 | -1.4 | -1.5 | -3.5 | -8.9 |
|  |  | CC | -0.2 | 0.4 | 1.5 | 1.1 | 1.2 | 1.6 | 0.9 | 1.2 | 2.1 | 1.6 | 1.0 | -0.2 |
| Itanki | Weather Station | Tx | 0.3 | 0.7 | 4.0 | 9.8 | 14.4 | 17.7 | 20.9 | 23.1 | 21.1 | 15.8 | 9.1 | 2.9 |
|  |  | RH | 69.8 | 70.1 | 70.7 | 73.7 | 78.7 | 86.2 | 89.2 | 88.4 | 80.7 | 72.1 | 68.6 | 68.6 |
|  |  | CC | 7.8 | 7.3 | 6.5 | 6.2 | 6.9 | 7.7 | 8.3 | 7.9 | 6.9 | 6.1 | 7.2 | 8.0 |
|  | GCM | Tx | 1.6 | 1.9 | 4.9 | 10.3 | 15.8 | 20.3 | 23.9 | 25.0 | 22.1 | 15.9 | 9.4 | 4.2 |
|  |  | RH | 87.0 | 85.2 | 80.9 | 79.3 | 81.1 | 86.6 | 89.3 | 88.0 | 83.0 | 79.2 | 80.7 | 85.2 |
|  |  | CC | 8.2 | 8.0 | 7.2 | 6.1 | 6.0 | 6.4 | 6.8 | 6.3 | 5.3 | 5.4 | 6.9 | 8.0 |
|  | Difference | Tx | -1.3 | -1.2 | -0.8 | -0.5 | -1.4 | -2.6 | -3.0 | -2.0 | -1.0 | -0.1 | -0.3 | -1.3 |
|  |  | RH | -17.3 | -15.2 | -10.2 | -5.6 | -2.5 | -0.3 | -0.1 | 0.4 | -2.3 | -7.1 | -12.0 | -16.5 |
|  |  | CC | -0.4 | -0.7 | -0.8 | 0.1 | 0.9 | 1.3 | 1.6 | 1.6 | 1.6 | 0.7 | 0.3 | 0.0 |
| Tatadohama | Weather Station | Tx | 11.1 | 11.1 | 13.6 | 17.7 | 20.6 | 23.2 | 26.6 | 28.4 | 26.0 | 21.8 | 17.6 | 13.6 |
|  |  | RH | 59.4 | 59.6 | 65.6 | 71.2 | 78.0 | 84.1 | 86.5 | 84.2 | 78.8 | 70.8 | 65.4 | 60.8 |
|  |  | CC | 4.8 | 5.4 | 6.4 | 6.4 | 7.3 | 8.1 | 7.7 | 6.1 | 6.9 | 6.2 | 5.4 | 4.4 |
|  | GCM | Tx | 8.8 | 9.1 | 11.8 | 16.1 | 20.0 | 23.3 | 25.9 | 26.9 | 25.1 | 20.4 | 15.5 | 11.2 |
|  |  | RH | 75.9 | 74.6 | 74.4 | 77.2 | 81.2 | 87.7 | 88.5 | 86.0 | 83.2 | 78.1 | 76.8 | 76.8 |
|  |  | CC | 4.8 | 5.0 | 5.2 | 5.7 | 6.2 | 7.2 | 7.0 | 5.9 | 5.6 | 4.8 | 4.4 | 4.6 |
|  | Difference | Tx | 2.3 | 2.0 | 1.8 | 1.6 | 0.6 | 0.0 | 0.6 | 1.5 | 1.0 | 1.4 | 2.1 | 2.4 |
|  |  | RH | -16.4 | -14.9 | -8.8 | -6.0 | -3.2 | -3.6 | -2.0 | -1.8 | -4.4 | -7.3 | -11.4 | -16.0 |
|  |  | CC | 0.0 | 0.4 | 1.1 | 0.6 | 1.0 | 0.9 | 0.7 | 0.2 | 1.3 | 1.4 | 1.0 | -0.2 |
| Yonehara | Weather Station | Tx | 21.3 | 21.5 | 23.3 | 25.9 | 28.3 | 30.4 | 32.0 | 31.8 | 30.6 | 28.4 | 25.9 | 22.9 |
|  |  | RH | 71.1 | 72.9 | 75.5 | 77.8 | 79.1 | 80.6 | 76.3 | 76.6 | 75.3 | 72.2 | 71.9 | 69.3 |
|  |  | CC | 8.1 | 8.0 | 7.8 | 7.6 | 7.6 | 7.1 | 6.1 | 6.2 | 6.4 | 6.6 | 7.2 | 7.7 |
|  | GCM | Tx | 19.2 | 19.5 | 21.2 | 23.4 | 25.6 | 27.2 | 28.0 | 28.2 | 27.5 | 25.7 | 23.3 | 20.8 |
|  |  | RH | 76.9 | 76.9 | 79.4 | 82.6 | 84.0 | 85.1 | 84.1 | 83.1 | 81.5 | 80.2 | 78.8 | 77.9 |
|  |  | CC | 6.1 | 5.7 | 5.4 | 5.4 | 5.9 | 6.3 | 6.0 | 5.8 | 5.1 | 5.5 | 6.0 | 6.4 |
|  | Difference | Tx | 2.1 | 2.1 | 2.1 | 2.5 | 2.7 | 3.2 | 4.0 | 3.6 | 3.1 | 2.8 | 2.5 | 2.1 |
|  |  | RH | -5.8 | -4.0 | -3.9 | -4.8 | -4.9 | -4.5 | -7.8 | -6.4 | -6.2 | -8.0 | -6.9 | -8.5 |
|  |  | CC | 1.9 | 2.3 | 2.4 | 2.2 | 1.7 | 0.9 | 0.1 | 0.4 | 1.3 | 1.2 | 1.2 | 1.2 |
