# Supplementary material for: Comparing future climatic suitability to shoreline loss for recreational beach use: a case study of five Japanese beaches
Source: Reg Environ Change. 2022 Mar 28;22(2):54. doi: 10.1007/s10113-022-01906-2 (PMC8958812; doi:10.1007/s10113-022-01906-2)
Supplement: Supplementary file 2 — (DOCX 15.6 kb) [file 10113_2022_1906_MOESM2_ESM.docx]

Table 1-Models utilized from the CMIP5 multi-model ensembles to project HCI:Beach scores and sea level rise.

| ***Institute ID*** | ***Modelling Group*** | ***Model Version Used*** | ***Used in HCI:Beach Projections and model range comparisons*** | ***Used for sea level rise ensembles (Figure 6)*** |
| --- | --- | --- | --- | --- |
| *CCCMA* | *Canadian Centre for Climate Modelling and Analysis* | *CanESM2* | *X* | *X* |
| *CNRM-CERFACS* | *Centre National de Recherches Meteorologiques/ Centre Europeen de Recherche et Formation Avancees en Calcul Scientifique* | *CNRM-CM5* | *X* | *X* |
| *CSIRO-QCCCE* | *Commonwealth Scientific and Industrial Research Organization in collaboration with Queensland Climate Change Centre of Excellence* | *CSIRO-Mk3.6.0* | *X* | *X* |
| *NOAA GFDL* | *Geophysical Fluid Dynamics Laboratory* | *GFDL-ESM2G* | *X* | *X* |
|  |  | *GFDL-ESM2M* | *X* | *X* |
| *NASA GISS* | *NASA Goddard Institute for Space Studies* | *GISS-E2-R* | *X* | *X* |
| *MOHC (additional realizations by INPE)* | *Met Office Hadley Centre (additional HadGEM2-ES realizations contributed by Instituto Nacional de Pesquisas Espaciais)* | *HadGEM2-ES* | *X* | *X* |
|  |  | *HadGEM2-CC* |  | *X* |
| *IPSL* | *Institut Pierre-Simon Laplace* | *IPSL-CM5A-LR* | *X* | *X* |
|  |  | *IPSL-CM5A-MR* | *X* | *X* |
| *MIROC* | *Atmosphere and Ocean Research Institute (The University of Tokyo), National Institute for Environmental Studies, and Japan Agency for Marine-Earth Science and Technology* | *MIROC5* | *X* | *X* |
| *MIROC* | *Japan Agency for Marine-Earth Science and Technology, Atmosphere and Ocean Research Institute (The University of Tokyo), and National Institute for Environmental Studies* | *MIROC-ESM* | *X* | *X* |
|  |  | *MIROC-ESM-CHEM* | *X* | *X* |
| *MRI* | *Meteorological Research Institute* | *MRI-CGCM3* | *X* | *X* |
| *NCC* | *Norwegian Climate Centre* | *NorESM1-M* | *X* | *X* |
|  |  | *NorESM1-ME* |  | *X* |
| *BCC* | *Beijing Climate Center, China Meteorological Administration* | *BCC-CSM1.1* |  | *X* |
| *CSIRO-BOM* | *CSIRO( Commonwealth Scientific and Industrial Research Organisation in collaboration with the Queensland Climate Change Centre of Excellence)* | *ACCESS 1.0* |  | *X* |
| *INM* | *Institute for Numerical Mathematics* | *INM-CM4* |  | *X* |
| *MPI-M* | *Max Planck Institute for Meteorology (MPI-M)* | *MPI-ESM-LR* |  | *X* |
|  |  | *MPI-ESM-MR* |  | *X* |
